# A simple, one-step polychromatic staining method for epoxy-embedded semithin tissue sections

**DOI:** 10.1093/jmicro/dfy037

**Published:** 2018-10-15

**Authors:** Shunichi Morikawa, Azusa Sato, Taichi Ezaki

**Affiliations:** 1 Department of Anatomy and Developmental Biology, Tokyo Women’s Medical University, 8-1 Kawada-Cho, Shinjuku, Tokyo, Japan; 2 Department of Chemistry, Tokyo Women’s Medical University, 8-1 Kawada-Cho, Shinjuku, Tokyo, Japan

**Keywords:** polychromatic staining, epoxy resin, semithin section, azure B, basic fuchsin, tetraborate, light microscopy

## Abstract

Although conventional toluidine blue staining is a common technique used for rapid observation of semithin sections prior to transmission electron microscopy, it is monochromatic and insufficient for accurate identification of different tissue components by light microscopy. Additionally, polychromatic staining methods generally require step-by-step processes involving different dyes, and it is often difficult to balance the color tone of each step. In this study, we developed a simple polychromatic staining method for epoxy-embedded tissue sections. We stained preheated sections with an aqueous ethanol solution of azure B and basic fuchsin, with the addition of sodium tetraborate to enhance the staining efficacy. We optimized various staining conditions to enable sufficient coloration easily and consistently in a single, rapid staining step, using a single staining-mixture solution. Our method enabled clear differentiation of various tissue structures according to color tone and stain intensity, thereby facilitating the detection of fine structural differences, including various organelle and inclusion bodies. This technique represents a simple polychrome-staining method to allow more informative and convincing histological investigation in various fields of research and education.

## Teaser

A simple polychromatic staining method for epoxy-embedded semi-thin tissue sections was developed to allow more informative and convincing histological investigation in various fields of research and education. This technique enabled clear differentiation of various tissue structures according to color tone and intensity, thereby facilitating the detection of fine structural differences.

## Introduction

Semithin sections of resin-embedded tissues are useful for identifying regions of interest that can subsequently be closely examined by transmission electron microscopy (TEM) and are thus considered a step in the preparation of TEM samples. However, due to their thinness (usually 0.5 μm) and the good morphological preservation achieved by treatment with reliable fixatives specifically prepared for TEM, epoxy semithin sections are highly valuable, as they enable much finer visualization of tissue elements than conventional paraffin sections usually fixed with formalin and at 3- to 5-μm thickness.

Toluidine blue is a standard dye used for semithin sections [1,2] that allows for rapid assessment of regions of interest prior to TEM observation. However, this is a monochromatic dye and is insufficient for close identification of different tissue components. Polychromatic staining, on the other hand, provides better histological contrast than monochromatic staining and would render semithin sections excellent materials for use in morphological research and education and to facilitate subsequent TEM examination. Various polychromatic staining techniques for epon-embedded semithin sections have been developed (Table 1). However, these methods usually require several staining steps and the preparation of numerous complicated reagents [3,4] or long incubation times [5–7]. These methods are thus generally not as simple to perform as single-step staining with toluidine blue. Additionally, when sections are stained using step-by-step methods, it is often difficult to balance the different color tones of the dyes used in each step [7,8], and a certain level of skill might be necessary to obtain consistent staining results [9,10]. Otherwise, the stain used in the last step often becomes predominant and overshadows the staining done in preceding steps [6,11]. It is also possible that the colors may begin to fade within a few days;[5] thus, the staining solutions may not remain stable and may need to be prepared fresh before each use [12,13].

**Table 1.** Comparison of various polychromatic staining techniques for Epon-embedded tissue sections

Reference Author [#]	Staining solution			Staining procedures			Special remarks
	Dyes^a^	Preparation	Stability	Steps^b^	Time required	Condition	
Grimley [5]	TB + MG/BF	Easy	Remove surface film	2(4–5)	Long (5–30 min)	Hot plate 70˚C	Fading occurs within several days
Aoki *et al.* [6]	TB/BF/LB	Complicated to adjust pH	ND^c^	3(8)	Long (~10 min)	Hot plate	pH adjustment is critical 90˚C Mounting medium is important
Huber *et al.* [12]	BF/MB	Very easy	BF: prepare fresh &filter	2(5)	Short (~5 min)	Hot plate 70˚C	Sections loosen under high pH 12.5 Oil mounting prevents stains from fading
Aparicio *et al.* [13]	MB/BF	Easy	A few days filter everyday	2(4)	Very short (~a few min)	Hot plate 70˚C	BF staining time is critical
Sato *et al.* [10]	MB + BF	Easy	Stable for several months	1(2)	Very short (4–5 s)	Burner	Higher pH gives more precipitation 45–50˚C
Humphrey *et al.* [11]	MB + AA/BF	Easy	Stable for several months	2(4)	Short (~5 min)	Hot plate 65˚C	Mounting medium is important
Jha [7]	MB + BF	Complicated to adjust pH	Stable for several months	1(4)	Long (~10 min)	Hot plate	Different results depending on age 52˚C of solutions
Rock *et al.* [8]	MB + AA/BF	Complicated	Stable for 4 months at 4˚C in the dark	2(4)	Very short (~a few min)	Warming block 50˚C	Staining vary with time and temperature
Tolivia *et al.* [3]	MB+GV + PY/PA+phenol	Complicated	ND	2(8)	Short (5–10 min)	Room temp.	Highly sensitive to osmication intensity
D’Amico [9]	MB + AB/BF	Easy	Stable with a neutral pH	2(4)	Very short (2 min)	Flame	Staining time is critical Heating can destain sections
Relucenti *et al.* [4]	MB + AB/BF/AR	Complicated	Stable for several months in the dark	3(7)	Very short (~a few min)	Hot plate 65˚C	Suitable for pathological samples
Morikawa *et al.* (Current study)	AB + BF	Very easy	Stable for 5 years at room temp. in the dark	1(2)	Very short (~60 s)	Hot plate	Timing of borax addition is critical 100˚C

[#]: Reference number.

^a^AR: Alizarin red, AA: Azure A (or Azure II), AB: Azure B (Azure I), BF: Basic fuchsin, GV: Gentian violet, LG: Light green, MG: Malachite green, MB: Methylene blue, PA: Pararosaniline, Pyridine: PY, TB: Toluidine blue.

^b^The numbers indicate required incubation steps with actual staining solutions (total staining steps including steps for washing of staining solutions).

^c^ND: No description.

Therefore, we aimed to improve conventional polychromatic staining techniques and develop a simple and rapid one-step method for coloring semithin sections that can be used similarly to conventional toluidine blue monochromatic staining. Our technique enabled clear differentiation of various tissue elements according to color contrast and intensities following optimization of the dye ratios in the staining solution to obtain maximum color differentiation of structures easily and consistently. Moreover, preparation of the staining solution was simple, and the effective coloration was consistent after one-step and within 3 min. The colors of the stained sections and staining solutions have remained stable for >5 years. Here, we describe the improved staining protocol for epoxy-embedded semithin tissue sections and discuss the possible staining mechanism, conditions required for optimal staining results, and the advantages of using our method for examining both normal and pathologic samples. Specimens prepared using our method may be useful for clinical research, as well as for the education of medical and paramedical students.

## Methods

### Animals

C57BL/6 and BALB/c mice (10–12-weeks old) were purchased from Japan SLC, Inc. (Shizuoka, Japan) and maintained in an air-filtered room at the Institute of Laboratory Animals, Tokyo Women’s Medical University (TWMU). Animals had access to sterilized standard laboratory chow and water *ad libitum*. The procedures employed for the handling and care of animals were approved by the Animal Experiment Committee, TWMU and experiments were performed in accordance with legislation of the Institute of Laboratory Animals for Animal Experimentation at TWMU. For anesthesia, intramuscular injection of ketamine (87 mg/kg body weight) plus xylazine (13 mg/kg body weight) was used.

### Preparation of pathological specimens

Pathological animal models (*n* ≥ 3 mice per group) were produced according to the procedures described below.

#### Wound-healing models

Experimental wounds were made according to the method described in our previous study [14]. Briefly, a 2-mm-diameter punch was used on anesthetized animals to make circular wounds on the dorsal skin, and the wounded areas were covered with a sheet of sterile gauze and allowed to heal. On the 5th day after wounding, animals were sacrificed for experimentation.

#### Tumor models

Lewis lung carcinoma cells [15] (American Type Culture Collection, Rockville, MD, USA) were implanted (2.5 × 10^6^ cells in 250 μL phosphate-buffered saline) under the dorsal skin of anesthetized animals. Animals were sacrificed for specimen collection when tumors had grown to ~10 mm in diameter, which usually occurred ~2 weeks after implantation.

#### Hepatic steatosis models

Experimental nonalcoholic hepatic steatosis was induced by feeding animals a methionine–choline-deficient (MCD) diet, as described previously [16]. Animals that had been fed an MCD diet for 30 days were sacrificed for experimentation.

#### Allergic dermatitis models

Allergic dermatitis was induced by repeated application of 2,4-dinitrofluorobenzene (DNFB) to the ears of mice [17]. The ears of animals were painted with 25 μL of 0.15% DNFB (v/v) dissolved in vehicle [acetone/olive oil (3:1)] once a week for 5 weeks. Animals were sacrificed for experiments 24 h after the 5th round of painting.

### Preparation of epoxy-embedded semithin sections

Under anesthesia, all normal and pathologic model mice were fixed by vascular perfusion of 4% paraformaldehyde and 2.5% glutaraldehyde in 0.1 M sodium phosphate buffer (pH 7.4) for 5 min. Immediately after perfusion, tissues were removed, cut into small pieces, and immersed in the same fixative solution for another 2 h or overnight at 4°C. After rinsing with distilled water (DW), specimens were dehydrated through a graded-concentration series of ethanol (EtOH) and embedded in epoxy resin (Epon 812 resin; TAAB Laboratories, Aldermaston, UK) according to a standard procedure [18,19] by using propylene oxide as the transitional solvent. Epoxy resin was polymerized horizontally in an oven at 60°C for two nights. Semithin sections (0.5 μm thick) were made using a Leica ultramicrotome (Leica Microsystems, Wetzlar, Germany) and mounted onto glass slides. Mounted sections were then dried on a hot plate.

For bone tissue (mouse cochlea), decalcification was performed after aldehyde fixation. Briefly, bone tissues were treated with 10% neutral EDTA (Wako Pure Chemical Industries, Tokyo, Japan) for 5 days at 4°C with gentle agitation. The EDTA solution was changed daily. After decalcification, tissues were embedded in epoxy resin using the same procedure described.

### Preparation of staining solutions

Azure B (C.I. 52010), basic fuchsin (C.I. 42510), and sodium tetraborate (borax) were purchased from Wako Pure Chemical Industries, Ltd. The following two solutions were prepared.

(A) Staining dye mixture solution: 0.035% azure B and 0.015% basic fuchsin in 5% aqueous EtOH. First, 0.05% solutions of azure B and basic fuchsin dissolved in of 5% EtOH were prepared, with the azure B and basic fuchsin solutions then mixed at a ratio of 7:3 (v/v), respectively.

(B) Borax solution: 0.1 M sodium tetraborate aqueous solution (w/v). A 0.1 M borax solution was prepared by dissolving 3.81 g of sodium tetraborate (Wako Pure Chemical Industries, Ltd) in DW to final volume of 100 mL.

These staining solutions remained stable without any filtration for at least 5 years at room temperature (usually ~20–25°C) in the dark.

The toluidine blue (C.I. 52040; Wako Pure Chemical Industries, Ltd) solution was also prepared as a 0.05% solution in 5% EtOH. Toluidine blue staining was performed according to the same protocol used for our staining method.

### Staining procedures for the standard staining protocol

The standard staining protocol consists of three steps, as follows:

- Borax solution (B) was added to the staining dye mixture solution (A) at a ratio of 1:5 (v/v) to make the final staining solution ≤20 min before staining.
- Glass slides with sections were placed on a hot plate and preheated at 100°C, and the staining solution was dropped onto the sections to cover the entire surface and incubated for 30 s at 100°C.
- The staining solution was removed by rinsing with DW. Water drops remaining on the glass slides were removed using a blower.

**Fig. 1.**
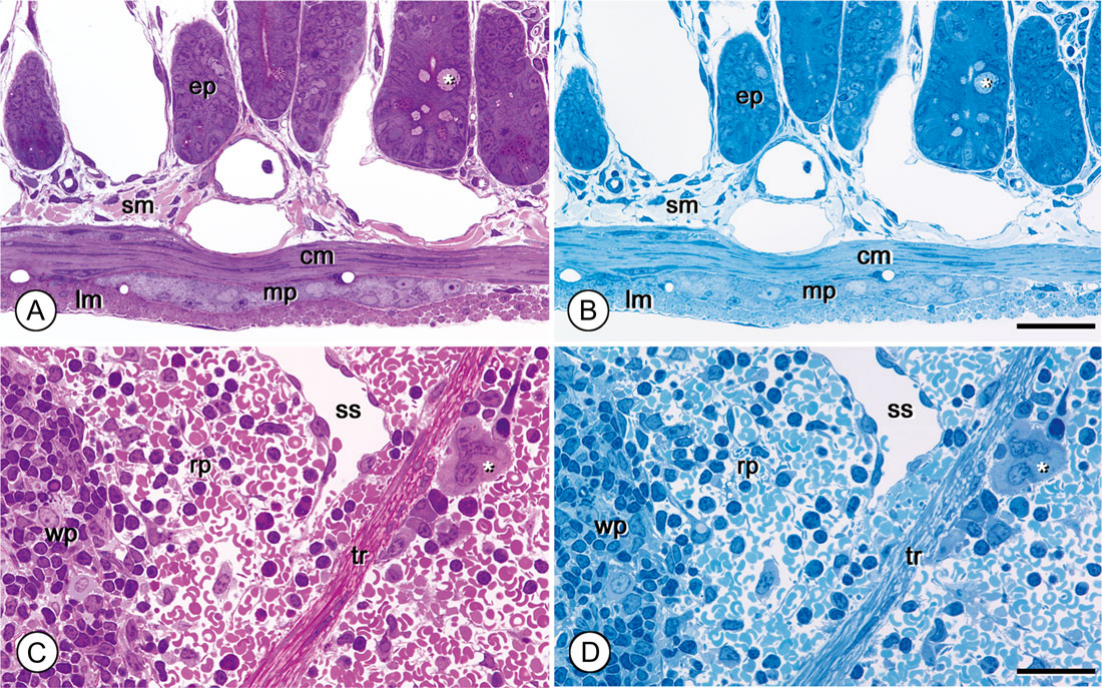
Original basic staining compared with routine toluidine blue single staining. Epoxy semithin sections of mouse jejunum (A) and spleen (C) stained by our original basic method. Adjacent serial sections of the jejunum and spleen (B & D, respectively) were stained with toluidine blue. The polychromatic effect in (A & C) adds histological contrast to each structure and gives more detailed information. ep: epithelium, sm: submucosa, cm: circular muscle layer, mp: myenteric plexus, lm: longitudinal muscle layer, asterisk (*) in A and B: goblet cell. wp: white pulp, rp: red pulp, ss: splenic sinus, tr: trabeculla, asterisk (*) in C and D: megakaryocyte. Magnifications are the same in all figures. Bar: 50 μm.

**Fig. 2.**
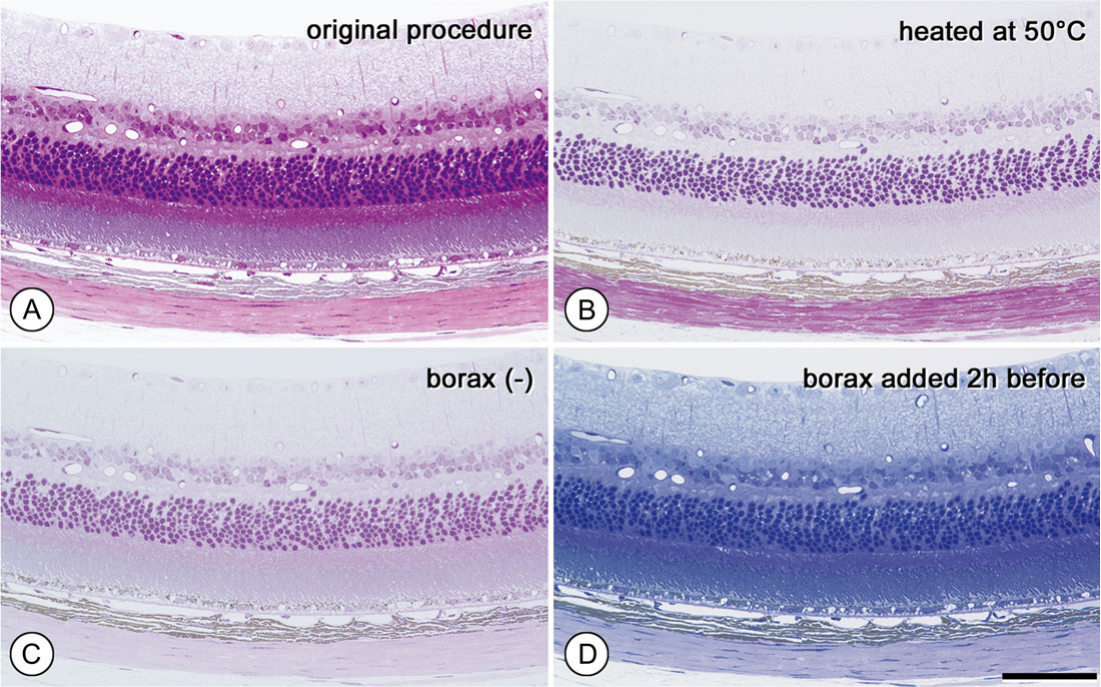
Verification of conditions in the staining procedure. Serial sections of retina. (A): Original procedure. Staining at 100°C with borax for 30 s. (B): Staining at 50°C. The stain is largely attenuated. (C): Staining without borax. (D): Staining with solution to which borax had been added 2 h before staining. Basic fuchsin stain is largely attenuated, but azure B remains intense. Magnifications are the same in all figures. Bar in E: 20 μm.

As an original basic staining protocol before establishing the described standard staining protocol, we used a saturated tetraborate aqueous solution (w/v) and a lower temperature (80°C) during staining on the hot plate (Fig. 1). As satellite experiments, we preliminarily tested various modified versions of the basic protocols, with each of the following three modified protocols differing from the original protocol in only one item. The modifications were as follows. (1) sections were stained at 50°C instead of 100°C for 30 s (Fig. 2B); (2) the addition of borax solution (B) to the staining dye mixture solution (A) was omitted (Fig. 2C); and (3) borax was added to the staining solution 2 hours before, rather than immediately before, staining (Fig. 2D).

**Fig. 3.**
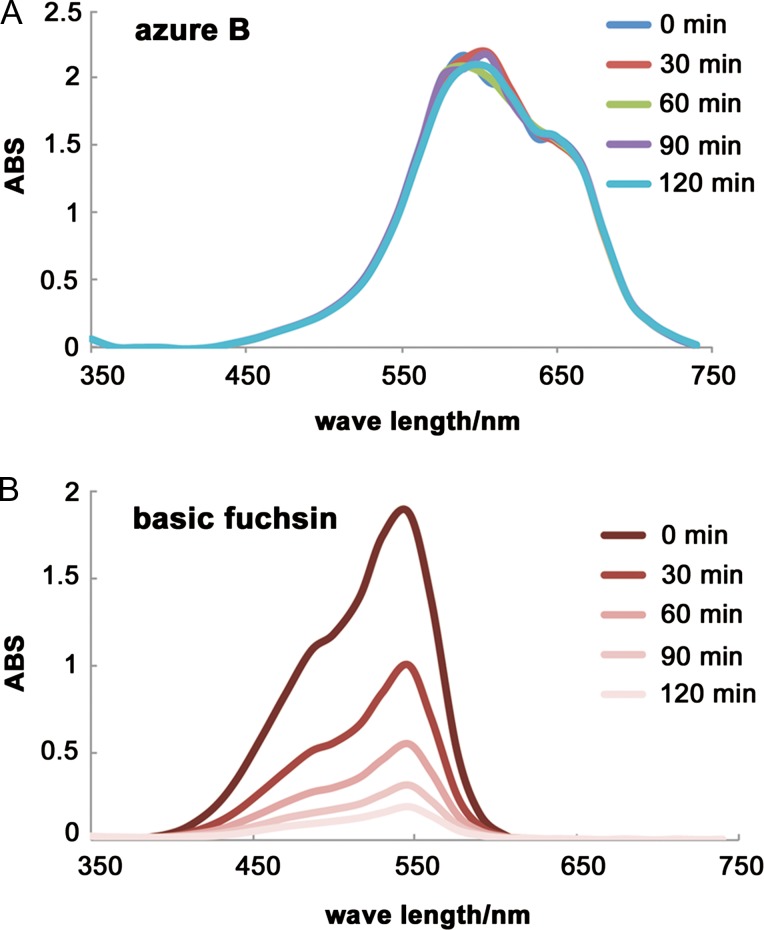
Time-dependent attenuation of basic fuchsin absorption spectrum after borax addition. Absorption spectra for azure B and basic fuchsin solutions after borax addition were measured at 0 min (immediately after borax addition), 30 min, 60 min, 90 min and 120 min. (A): The absorbance of azure B solution was nearly constant at all time points. (B): In contrast, the absorbance of the basic fuchsin solution was affected at as early as 30 min and gradually decreased with time after the addition of borax. The waveform of its absorption spectrum became nearly flat at 120 min. ABS: absorbance.

After staining, sections were mounted with the same pre-polymerized epoxy resin that had been aliquoted and stored in a deep freezer at ≤−30°C, cover slipped, and placed in an oven at 60°C overnight to allow the resin to harden. Although epoxy resin is the most suitable mounting medium in terms of refractive index, Permount (Thermo Fisher Scientific, Waltham, MA, USA) or similar mounting media can be used to accelerate the process. All sections were photographed using a Keyence BZ-9000 all-in-one microscope (Keyence Co., Osaka, Japan).

### Spectrophotometry

We examined time-dependent changes in the absorption spectra of azure B and basic fuchsin solutions after the addition of borax solution (B). Specifically, a 0.1-M borax solution was added to an aqueous solution containing either 0.035% azure B in 5% ethanol or 0.015% basic fuchsin in 5% EtOH. In both cases, the final concentration of the chromatic chemical and the final ratio of borax solution (B) to stain solution (A) was the same as that used in the original staining procedure. Absorption spectra of each dye solution were measured using a NanoDrop 2000C spectrometer (Thermo Fisher Scientific) at each of the following five time points: 0, 30, 60, 90 or 120 min after the addition of borax solutions, where 0 min indicates that the measurement was taken immediately after the addition of borax. All measurements were performed over a 350–740 nm range in a micro-volume-measuring chamber within the spectrometer (path length: 1.0 mm). The spectra of each sample were measured at 26°C, and data are expressed and presented as line graphs.

## Results

### Original basic staining conditions

As an original basic staining protocol for our method, various cell components and connective-tissue elements of epoxy-embedded semithin sections of the small intestine and spleen were preliminarily stained using a saturated borax solution on a hot plate at 80°C and compared with conventional toluidine blue staining. Our method highlighted additional structural details in different color tones and provided more information (Fig. 1A and C) as compared with toluidine blue single staining (Fig. 1B and D). In other preliminary experiments, color differentiation was optimal at a dye ratio of 7:3 (0.05% azure B solution: 0.05% basic fuchsin solution).

### Establishment of the standard protocol by altering staining conditions

To determine whether the original basic staining condition fulfilled our standard color differentiation for epon-semithin sections, we examined variations in the staining procedures. In the original basic staining procedure, a saturated borax solution was added to the staining dye mixture solution (A) immediately prior to the staining step, and the sections were stained at 100°C for 30 s (Fig. 2A).

In the first satellite experiment, the effect of temperature on the hot plate was tested at 50°C instead of 100°C for 30 s (Fig. 2B), with treatment at the lower temperature substantially reducing overall staining intensity. Increasing the temperature to 80°C resulted in staining intensity results still not comparable to those obtained at 100°C. However, at a higher temperature (150°C), the staining solution quickly evaporated, and the basic fuchsin stain became faint (data not shown). Therefore, subsequent staining procedures were performed at 100°C.

Next, we omitted addition of borax solution (B) to the staining dye mixture solution (A). We found that borax alkalization of the dye solution was essential to obtain the best color combination of both azure B and basic fuchsin, and that omission of borax resulted in a considerable decrease in color contrast (Fig. 2C). Additionally, we examined the effect of the borax solution by eliminating its addition to each respective staining solution. When sections were stained with each staining solution containing DW instead of borax, the intensity of both azure B and basic fuchsin staining was considerably weakened relative to that obtained using our original protocol (Fig. 2A). Specifically, azure B staining was largely lost in the absence of borax solution. Although the attenuated effects of borax omission were more marked in azure B than in basic fuchsin staining, the addition of borax enhanced basic fuchsin staining (data not shown).

We then assessed the timing of borax addition to the staining dye mixture solution. When borax was added exactly 2 h earlier, basic fuchsin staining was almost completely absent, whereas azure B staining remained intense and uniform (Fig. 2D). Furthermore, spectrophotometric measurements showed that the absorbance patterns were similar to those from the stained tissue sections. The absorbance of azure B solution was nearly constant at all time points (0, 30, 60, 90 or 120 min) following the addition of borax (Fig. 3A), whereas the absorbance of the basic fuchsin solution decreased drastically over time following the addition of borax (Fig. 3B). At 30 min after borax addition, basic fuchsin staining was attenuated; therefore, these results suggested that borax should be added to the staining mixture solution (A) within 20 min before staining.

### Characterization of various cellular elements

Based on these results, we fixed the staining procedures according to the standard staining protocol and assessed their effects on the staining characteristics of various biological structures. Results for both cellular and extracellular elements are summarized in Table 2.

**Table 2.** Summary of staining profiles of various tissue components.

Structures	Image color	Characteristics and notes
Cellular elements		
Nuclear chromatin	Dark purple to nearly black	c.f. Nucleoplasm is light pink to reddish purple (Figs 4–6).
Cytoplasm	Light to medium purple	Lighter than chromatin. Staining intensity and color tone vary considerably (Figs 4–6)
in erythrocytes	Red or deep pink	(Fig. 4B and C)
in polymorphonuclear leukocytes	Conspicuous red	(Figs 4C and D, 6D)
in muscle cells	Purple	(Figs 4E, 5A)
Myelin sheath	Nearly black	(^a^Figure not shown)
Mitochondria	Deep purple	Prominent in myocardial cells (Fig. 4E)
Ribosomes (plasma cells)	Weak red–purple	Distinct from juxtanuclear pale (Golgi) area^a^
Lysosomes (macrophages)	Red	Prominent in macrophages (Fig. 4B).
Lipid droplets	Gray blue	Leydig cells (Fig. 4A), adipose cells (Fig. 4F), sebaceous glands (Fig. 4G).
Alveolar surfactant	Gray blue	Similar staining to lipid droplets^a^
Mucus in goblet cells	Faint pink to medium purple	Staining varies even in a cross section of an intestinal wall (Fig. 4H).
in sublingual glands	Faint purple	Stained uniformly^a^
Zymogen granules in pancreatic acinar cells	Deep reddish purple	Stained denser tone in the cytoplasm^a^
Paneth cell granules	Deep reddish purple	Stained denser tone in the cytoplasm^a^
Mast cell granules	Dark blue–black	Stained darker than other granules (Fig. 4F)
Fibers		
Collagen fibers	Light pink	Fibers in general are preferentially stained with basic fuchsin. (collagen fibers; Figs 5A–C, 6A).
Elastic fibers	Red	Clearly distinguishable from collagen or reticular fibers (Fig. 5B and C)
Reticular fibers	Light pink	Similar to collagen fiber staining, but distinguishable by fineness of the fibers and locations (in reticular tissues; Fig. 5D)
Matrices		
Cartilage matrix	Deep reddish purple	Interterritorial matrix is stained with gradation of deep reddish purple (Fig. 5E).
Bone matrix	Light reddish pink	Similar to collagen fiber staining, but stained coarse reddish pink (Fig. 5F)

^a^Figure not shown.

**Fig. 4.**
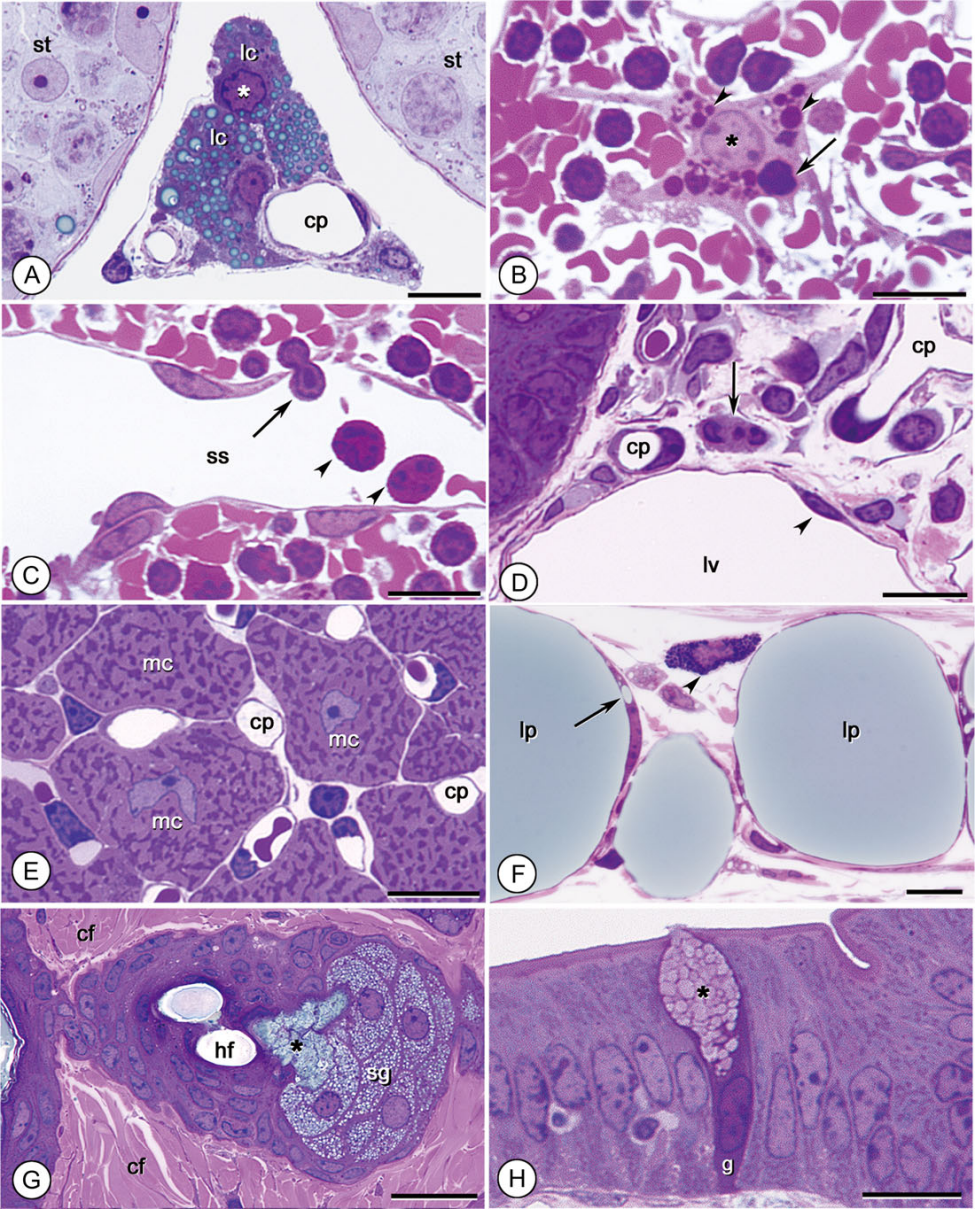
Appearance of cellular elements. (A): The testis. Leydig cells (lc) contain lipid droplets that stain gray-blue. Nuclear chromatin (*) stains denser than the cytoplasm. st: seminiferous tubules. cp: capillary. Bar: 10 μm. (B): Macrophage in the spleen. Lysosomes of various sizes stain red (arrowheads). A lymphocyte is captured within the cell (arrow). Bar: 10 μm. (C): The splenic sinus (ss). Neutrophils (arrowheads) have red-stained cytoplasm. A lymphocyte (arrow) is migrating through the endothelium. Bar: 10 μm. (D): The jejunum. A neutrophil (arrow) has a medium purple cytoplasm. cp: capillary. lv: lymphatic vessel. Bar: 10 μm. (E): Heart. Numerous mitochondria of various cross-sectioned shapes stain deep purple and are tightly packed in myocardial cells (mc). cp: capillary. Bar: 10 μm. (F): The subcutis. Adipose cells contain gray-blue-stained large lipid droplets (lp). A tiny conjunctive lipid droplet (arrow) formed next to a large droplet. The granules of a mast cell (arrowhead) are stained ultramarine blue or nearly black. Bar: 20 μm. (G): The dermis. Sebaceous glandular cells (sg) contain numerous lipid-rich sebum vesicles that are stained gray-blue. In the region next to the pore of the hair follicle (hf), sebum vesicles merge and form a lump of sebum (*). cf: collagen fibers. Bar: 20 μm. (H): Cytoplasm of a goblet cell (g) in the jejunum is stained deeper reddish purple than neighboring absorptive epithelial cells. Mucus-containing secretory granules (*) appear faint pink. Bar: 10 μm.

In general, the nucleus was readily recognizable as chromatin consistently stained much darker than the cytoplasm. The chromatin stained dark purple to nearly black, the nucleoplasm stained light pink to reddish purple, and the cytoplasm stained light to medium purple (Fig. 4A–H). However, staining intensity differed between tissues, as did the tones associated with the cytoplasm and nucleus. The cytoplasm of macrophages or sinus endothelia in the spleen appeared paler than those in other tissues (Fig. 4B and C). Furthermore, the cytoplasm of erythrocytes stained red or deep pink (Fig. 4B and C), while that of polymorphonuclear leukocytes was characterized by a conspicuous red tone (Fig. 4C and D). By contrast, the cytoplasm of myocardial cells appeared purple (Fig. 4E).

Among cell organelles and inclusion bodies, mitochondria stained deep purple, allowing their easy observation in cells (Fig. 4E). Additionally, the ribosome-rich cytoplasm of plasma cells was stained a relatively weak red-purple along with a distinct juxtanuclear pale area (data not shown). Lysosomes in macrophages tended to stain red (Fig. 4B). By contrast, lipid droplets in Leydig cells and fat cells appeared gray-blue, as did sebum vesicles in sebaceous cells (Fig. 4A, F and G). Granules of mast cells rich in histamine and heparin appeared dark blue to nearly black (Fig. 4F). Mucous vesicles in goblet cells in the small intestine were not strongly stained and appeared faintly pink to white (Fig. 4H), although their cytoplasm showed denser staining than that of macrophages (Fig. 4B).

### Characterization of various extracellular elements

**Fig. 5.**
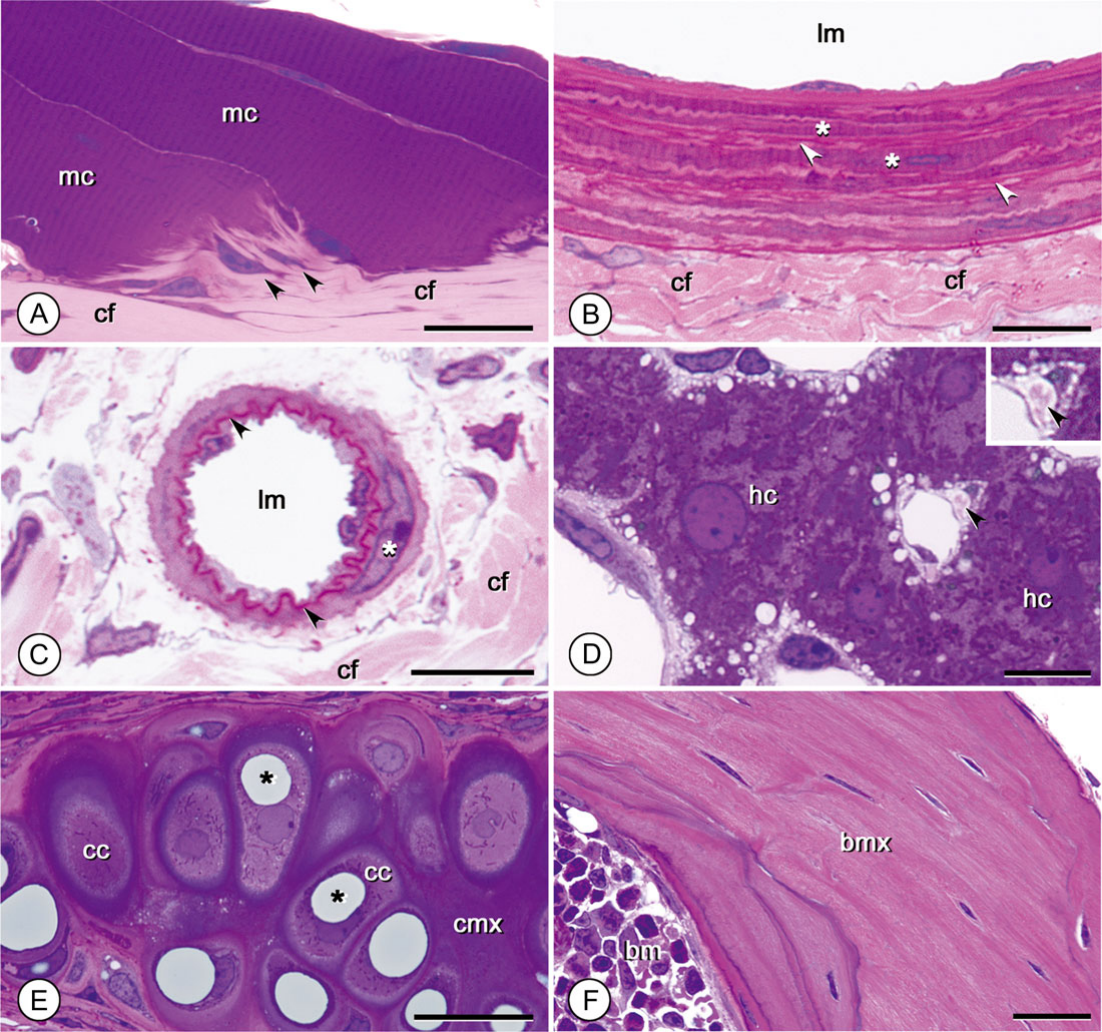
Appearance of extracellular elements. (A): Junction between the soleus muscle and the calcaneal tendon. Skeletal muscle cells (mc) stain purple, while collagen fibers (cf) appear pink. Fibroblasts accumulate at the junction (arrowheads) presumably to connect the muscles and the tendon strongly by collagen secretion. Bar: 10 μm. (B): The aorta. In the tunica media, red-stained elastic fibers (arrowheads) are recognizable in between smooth muscles (*), while collagen fibers (cf) in the tunica adventitia stain pink. lm: lumen. Bar: 10 μm. (C): An arteriole in the jejunum. Red-stained elastic fibers with a wavy pattern are highlighted (arrowheads). cf: collagen fibers, lm: lumen. (*): smooth muscle cell. Bar: 10 μm. (D): The liver. Around a sinusoid, thin reticular fibers stained pink can be observed [arrowheads; see also inset at higher magnification (×2 of the main figure)]. Note that the lipid droplets in hepatocytes tended to be small in size (~1 μm in diameter), whereas medium-sized droplets (~2–4 μm in diameter) were found in hepatic stellate cells (Ito cells) located in the space of Disse. hc: hepatocytes. Bar: 10 μm. (E): Hyaline cartilage of the trachea. Cartilage matrix (cmx) stains deep purple. The cytoplasm of cartilage cells stains medium purple. Many cartilage cells (cc) have gray-blue lipid droplets (*). Bar: 20 μm. (E): Cochlear bone. Bone matrix (bmx) stains dense pink. bm: bone marrow. Bar: 20 μm.

Connective-tissue fibers, such as collagen and elastic and reticular fibers, were preferentially stained with basic fuchsin rather than azure B, appearing pink or red and making them easy to distinguish from cellular structures (Fig. 5A–D). The fiber types were also distinguishable by their color intensity or thickness. Specifically, elastic fibers were intensely stained with basic fuchsin and appeared red (Fig. 5B and C), whereas collagen fibers (Figs 4G and 5A–C) and the finer reticular fibers (Fig. 5D) stained pink. In skeletal muscle-tendon junctions, muscle cells appeared reddish purple and were clearly distinguishable from the collagen fibers of the tendons, which appeared pink (Fig. 5A). Matrices of cartilage and bone tissue showed contrasting staining, with the matrix of hyaline cartilage from the trachea or elastic cartilage staining deep purple (Fig. 5E) and bone tissues staining a coarse reddish pink (Fig. 5F).

### Staining of pathologic tissues

**Fig. 6.**
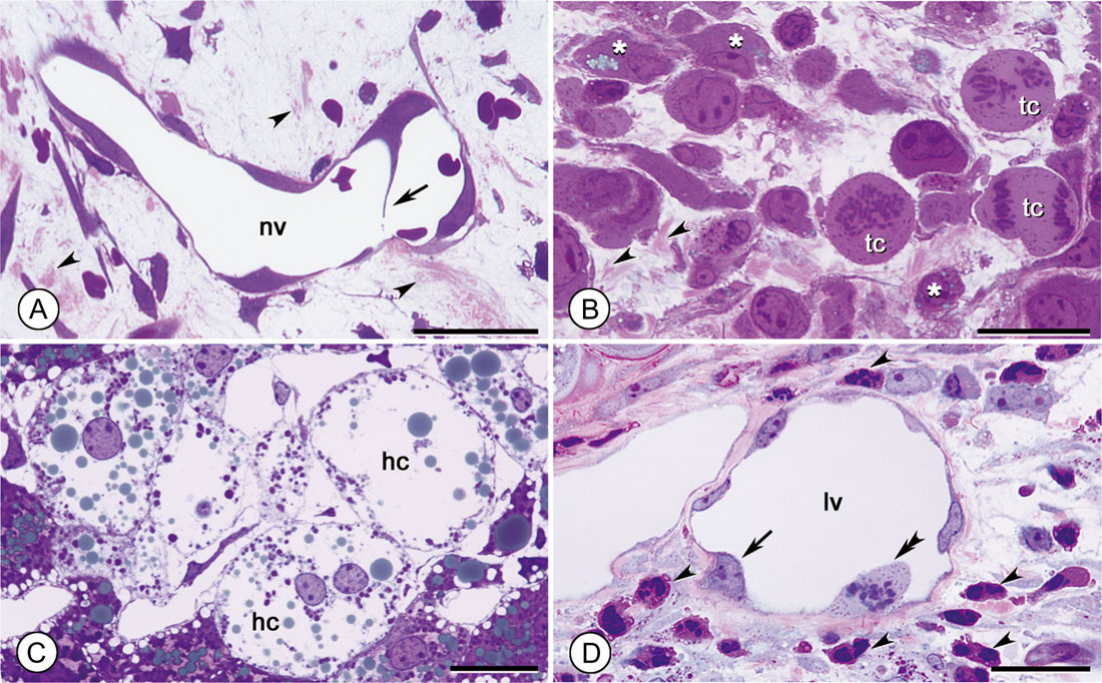
Appearance of pathological tissues. (A): Granulating tissue formed 5 days after the skin punching injury. Nascent fine collagen fibers (arrowheads) are identifiable by pink staining. A newly formed vessel (nv) can be detected. In the vessel, an endothelial cell inwardly projects a slender cytoplasmic process (arrow) to the opposite luminal surface, presumably to increase the number of vessels by splitting the lumen in the angiogenic process. Bar: 20 μm. (B): Lewis lung carcinoma. Deposition of fine nascent collagen fibers is seen in the stroma (arrowheads). Proliferating tumor cells (tc) with large oval cell bodies isolated from the tumor mass are also seen. Note that stromal cells (*) contain very small blue-gray lipid droplets. Bar: 30 μm. (C): Hepatic steatosis induced using a methionine and choline-deficient diet. Lipid droplets in swollen hepatocytes (hc) show a considerable range of sizes, and very small lipid droplets are identifiable. Bar: 10 μm. (D): Allergic dermatitis induced by the application of DNFB to the ears of mice. Red-stained inflammatory cells distribute to the edematous dermis (arrowheads). In a dilated lymphatic vessel (lv), an endothelial soma is inflated (arrow). An endothelial cell (double arrowheads) is undergoing mitosis, possibly to promote luminal enlargement for effective drainage of tissue fluid. Bar: 20 μm.

Based on the results from normal tissues, we examined several tissue samples obtained under various pathological conditions. On the 5th day after skin wounding of mice, we observed actively forming granulation tissue and newly forming blood vessels in the wound area. The distribution of nascent, fine collagen fibers was evident in the granulated tissue (Fig. 6A). Remarkably, in some newly forming vessels identifiable by their immature and extremely thin vessel walls, endothelial cells projected cellular processes into the vessel lumen (Fig. 6A). These projections presumably served to split the vessel lumen and consequently form new branches during the angiogenic process, as reported previously [20,21].

Deposition of nascent, fine collagen fibers which stained pink was also identified in the stroma of Lewis lung carcinomas (Fig. 6B). Moreover, our staining method allowed visualization of minute blue-gray lipid droplets (≤~1 μm in diameter) in some stromal cells in the tumors (Fig. 6B), with these stromal cells morphologically distinct from typical adipocytes.

In contrast to normal hepatocytes frequently containing relatively few and small blue-gray lipid droplets (~1 μm in diameter) (Fig. 5D), hepatocytes contained many medium- to large-sized lipid droplets (~5–10 μm in diameter) in nonalcoholic hepatic steatosis livers (Fig. 6C). Hepatocellular ballooning, a key feature of the disease [22], was also clearly identified. In the ballooned hepatocytes, most cellular contents were absent, and only lipid droplets and nuclei remained (Fig. 6C).

In allergic dermatitis in the ear induced by DNFB, numerous dilated lymphatic vessels were identified (Fig. 6D). These showed the general characteristics of lymphatic vessels, including thin walls and the absence of mural cells on the abluminal side, which was easily recognized. In the inflamed condition, lumens were conspicuously dilated, the stomata of lymphatic endothelial cells were often swollen, and some of the endothelial cells were undergoing mitosis (Fig. 6D).

## Discussion

The staining method described in this study is as simple as conventional toluidine blue monochromatic staining; however, the result is more informative due to its polychrome effect. Our method differs from previous polychromatic staining methods, which usually consist of multi-step staining processes (Table 1), by enabling sufficient and reproducible coloration following rapid, one-step staining. The several advantages of the technique described here over previous polychromatic staining procedures are summarized in Table 1.

One of the greatest advantages of our method in terms of practical use is that high-quality polychromatic staining of epoxy-embedded semithin tissue sections can be achieved rapidly and easily through a single-step staining procedure requiring only 3 min. Additionally, mordants and differentiation processes are unnecessary, and the color tones of the staining allow for easier discrimination of tissue structures relative to previous polychromatic staining methods. Our method provides reproducible and consistent results, unlike other step-by-step staining methods that require accurate balancing of the different color tones of the dyes used at each step. Furthermore, our method outperforms other techniques in terms of the conservation of stained sections [6,11,12] and staining solutions [12,13]. Sections stained using our method have not faded over the course of multiple years, and the staining solutions have also remained stable for >5 years at room temperature in the dark. Our staining method can also be applied to specimens embedded in other types of resins, such as Fluka water-soluble Durcupan, Quetol 651, Spurr, and Durcupan ACM (data not shown).

In contrast to routine hematoxylin and eosin (H–E) staining [23] and azan staining [24], our method uses two basic aniline dyes of similar molecular weight (305.83 kDa for azure B and 337.86 kDa for basic fuchsin) to differentiate tissue structures, with both dyes capable of sufficiently depicting overall tissue structures when applied separately, similar to toluidine blue (data not shown). However, these dyes likely differ in their affinity for a specific type of substance (e.g. a protein or saccharide) or tissue structure; therefore, when they are applied together at a certain ratio, a balance of differing affinities presumably yields a particular color tone. Additionally, the amount of the substance would also affect the staining intensity. Here, we observed optimal color differentiation at a dye ratio of 7:3 (azure B:basic fuchsin). By varying the dye concentrations, we found that a 10-fold concentration (0.35% azure B and 0.15% basic fuchsin) yielded the same result as that of the optimized method. Therefore, the lower concentration containing borax was determined as sufficient to achieve good staining results.

Heat treatment at a high temperature (100°C) is a key factor for maximal staining, with treatment at a lower temperature (~80°C) or a higher temperature (150°C) substantially reducing staining intensity. We found that heat treatment at ~80–100°C provided consistently good results.

Borax alkalization of the dye solution was another key factor for efficient staining, with omission of borax resulting in a considerable decrease in staining intensity. Although this decrease was more obvious in azure B than in basic fuchsin staining, borax addition enhanced basic fuchsin staining. Alkalization facilitates dye penetration into resin-embedded materials [12,19], and previous studies suggested the importance of staining-solution alkalinity in staining epon-embedded sections [12,25]. The addition of 0.1 M borax solution (B) at a ratio of 1:5 (v/v) increased the pH of the staining solution from 4.3 to 9.3, which was sufficient to obtain optimal color differentiation. When borax solution was added at 50% of the total volume (v/v), the pH did not increase beyond 9.3, and staining results were unchanged (data not shown).

The timing of borax addition was also critical. Interestingly, borax attenuated basic fuchsin staining in a time-dependent manner, as demonstrated by the staining conditions and spectrophotometric results showing a time-dependent attenuation of the absorbance of basic fuchsin following borax addition. Basic fuchsin staining was evidently weakened after 30 min following borax addition and continued to weaken through 120 min. Additionally, we confirmed no appreciable changes in basic fuchsin staining within 20 min after borax addition; therefore, borax should be added to the staining solution no later than 20 min before staining.

### Staining characteristics of cellular structures

The color and staining intensity of cellular structures varied, especially in the cytoplasm, with these variations possibly reflecting particular physiological states or functional roles. Intriguingly, leukocytes found in the spleen, bone marrow or inflamed tissues showed a unique red cytoplasmic staining. The color of leukocytes in these tissues might reflect the immunologically activated state of the cells. Lysosomes found in macrophages and the cell bodies of neutrophils appeared red. Basic fuchsin might have a stronger affinity for lysosomal contents, including acid phosphatases, and this color tone is likely caused by the affinity of these structures for basic fuchsin. For neutrophils, although their stainability differs from that of H–E staining of paraffin sections, this characteristic could be useful in certain situations purposes. For example, identification of an inflammatory response in target tissue would be easier in specimens prepared using our method than in those prepared using other methods, because this would enable the neutrophils to be readily identified by their red appearance, even at low magnification.

The deep purple appearance of the mitochondria was likely produced by similar affinities of both dyes for the constituents. When both dyes were used individually (not as a mixture), each dye clearly depicted mitochondria (personal observation). Toluidine blue, a basic aniline dye similar to azure B or basic fuchsin, also stains mitochondria in epoxy-embedded tissues [26,27]. A similar protocol could potentially be applied for staining mast cell granules with highly basophilic substances (i.e. histamine, heparin and chondroitin sulfate). It is possible that the binding of the dyes to these acid mucopolysaccharides (MPSs) yielded a dark color. Toluidine blue is also a good marker for glycosaminoglycans (GAGs), with this affinity previously detected at an ultrastructural level [28]. Acid MPSs are compactly packed in mast cell granules and would, therefore, be stained much darker than those in the cartilage matrix.

In mucus, the dyes presumably bound to the saccharide chains of GAG, a major constituent of mucus. Many types of saccharide molecules form branching short chains in GAGs or repeat disaccharide units that form long linear chains [29,30]. Furthermore, branching sugar chains exhibit various patterns, even within a single mucin molecule, and the sugar-chain patterns of mucins differ between salivary glands and goblet cells of the small intestine [29]. Goblet cells were characterized by deep purple-stained cell bodies, with the densely packed rough endoplasmic reticulum in their cell bodies [31] presumably responsible for this basophilicity. Accordingly, goblet cells appear to stain deeply when treated with other basic dyes, such as iron hematoxylin [32] or toluidine blue (data not shown).

Lipids and sebaceous vesicles typically appeared blue-gray, whereas lipid droplets and lipid-rich structures, such as the granules of type II alveolar cells, appeared gray blue, suggesting that azure B likely has a higher affinity for lipids. Because lipids were tinged gray due to osmification prior to staining, the gray blue color is likely produced by superposition of azure B blue on the osmium gray. The precise mechanism producing this distinct color tone remains unclear; however, this distinct color tone allowed clear visualization of even the extremely tiny lipid droplets (<1 μm) stored in non-adipocyte stromal cells in Lewis lung tumors from specimens prepared using our method.

### Staining characteristics of extracellular elements

One of the benefits of these highly differentiated color tones was the clear demarcation of connective-tissue fibers. Basic fuchsin preferentially stained extracellular fibers, with elastic fibers clearly distinguished from others based on their denser reddish staining. The affinity of basic fuchsin appeared stronger for elastin, the main component protein of elastic fibers, as compared with that for major collagen (type I collagen) or reticular fibers (mainly type III collagen). Type I and III collagens are similar in terms of their molecular composition and triple helix structure [33], and basic fuchsin presumably has a similar affinity for these fibers, resulting in a similar staining color. Basic fuchsin staining represents a good marker for collagen fibers and is used as the sole marker of collagen fibers in many preparations of dental tissues [34]. By contrast, elastic fibers found in arterial walls were characterized by distinctive red staining and readily differentiated from other connective-tissue elements. It is worth noting that basic fuchsin is usually combined with resorcin or aldehyde to stain elastic fibers in paraffin sections [24]. In our method, it allowed visualization of the same fibers in the absence of these additives. Our results suggested that this red staining was due to the strong affinity of basic fuchsin with the elastic fibers, which was comparable to results obtained using conventional Weigert’s resorcin-fuchsin solution and Elastica van Gieson staining.

Cartilage and bone matrix stained with considerably different colors. The dense purple staining of the cartilage matrix was likely caused by its abundant acid MPSs, and the dense pink appearance of bone matrix likely resulted from the preferential binding of basic fuchsin to the type I collagens in the matrix. Decalcification with EDTA might also yield denser staining relative to that obtained from the collagen fibers in connective tissue.

### Application of the staining method to pathologic tissues

Our multi-color staining technique and the thinness of the epoxy semithin sections simplified identification of subtle pathological changes in various experimental models. For example, the high-quality labeling of collagen fibers might be of great help in identifying initial fibrosis associated with particular diseases, as well as in detecting *de novo* formation of connective tissue in specimens from early developmental stages. The ability of our method to distinguish between activated or nascent leukocytes and normal leukocytes could also be useful for examining pathologic or developmental tissue samples, although this capacity must be carefully examined in further studies in combination with immunohistochemical approaches.

## Concluding Remarks

Our method allowed for simple and rapid polychromic visualization of fine structures in epoxy-embedded semithin tissue sections. This method enabled visualization of both collagen and elastic fibers, particular cell types and specific intracellular structures in distinct color tones and could be highly useful for studies of both normal tissues and developmentally staged specimens and pathologic tissues in various fields of biomedical research and education.
